# Assessment of educational technology in lactation physiology by health students

**DOI:** 10.1590/0034-7167-2023-0252

**Published:** 2024-05-27

**Authors:** Daiani Oliveira Cherubim, Polyana de Lima Ribeiro, Tassiane Ferreira Langendorf, Cristiane Cardoso de Paula, Stela Maris de Mello Padoin

**Affiliations:** I Universidade Federal de Santa Maria. Santa Maria, Rio Grande do Sul, Brazil

**Keywords:** Educational Technology, Translational Biomedical Sciences, Nursing, Health Sciences Students, Biomedical Technology Assessment, Tecnologia Educacional, Ciencia Traslacional Biomédica, Enfermería, Estudiantes del Área de la Salud, Evaluación de la Tecnología Biomédica, Tecnologia Educacional, Ciência Translacional Biomédica, Enfermagem, Estudantes de Ciências da Saúde, Avaliação da Tecnologia Biomédica

## Abstract

**Objectives:**

to assess the suitability, facilitators, and barriers of using a video clip for teaching lactation physiology to health students.

**Methods:**

a cross-sectional study was conducted with online data collection at a higher education institution, using the Assistive Technology Assessment Instrument and open-ended questions. The sample consisted of 88 students.

**Results:**

the video clip was deemed suitable in all attributes. Facilitators identified included attractiveness, musicality, and ease of access. Barriers noted were the music’s speed and the necessity for prior knowledge. The video clip achieved adequate scores for interactivity (1.71), purpose (1.77), relevance (1.64), and clarity (1.77). The overall average of the attributes was 1.72.

**Conclusions:**

the video clip can serve as an effective learning strategy to enhance hybrid education, potentially contributing to the promotion and support of breastfeeding. However, some barriers underscore the importance of prior knowledge for a complete understanding of the content.

## RESUMO

**Objetivos:**

avaliar a adequação, facilitadores e barreiras para o uso de um videoclipe para aprendizagem da fisiologia da lactação por estudantes da saúde.

**Métodos:**

estudo transversal com coleta de dados online em uma instituição de ensino superior, utilizando o Instrumento de Avaliação de Tecnologia Assistiva e questões abertas. A amostra incluiu 88 estudantes.

**Resultados:**

o videoclipe foi avaliado como adequado em todos os atributos. Facilitadores identificados incluíram atratividade, musicalidade e praticidade de acesso. Barreiras apontadas foram a velocidade da música e a necessidade de conhecimento prévio. O videoclipe obteve média adequada para interatividade (1,71), objetivo (1,77), relevância (1,64) e clareza (1,77). A média global dos atributos foi 1,72.

**Conclusões:**

o videoclipe pode ser utilizado como estratégia de aprendizagem que potencializa o ensino híbrido na formação, com potencial para contribuir com a promoção e apoio ao aleitamento materno. No entanto, algumas barreiras destacam a necessidade de conhecimento prévio para a compreensão total do conteúdo.

## RESUMEN

**Objetivos:**

evaluar la idoneidad, los facilitadores y las barreras del uso de un videoclip para enseñar fisiologia de la lactancia a estudiantes de salud.

**Métodos:**

se realizo un estudio transversal con recopilación de datos en línea en una institución de educación superior, utilizando el Instrumento de Evaluación de Tecnologia Asistiva y preguntas abiertas. La muestra consistió en 88 estudiantes.

**Resultados:**

el videoclip fue considerado adecuado en todos los atributos. Los facilitadores identificados incluyeron atractivo, musicalidad y facilidad de acceso. Las barreras señaladas fueron la velocidad de la música y la necesidad de conocimientos previos. El videoclip obtuvo puntuaciones adecuadas en interactividad (1.71), propósito (1.77), relevancia (1.64) y claridad (1.77). El promedio general de los atributos fue de 1.72.

**Conclusiones:**

el videoclip puede servir como una estrategia de aprendizaje efectiva para mejorar la educación híbrida, potencialmente contribuyendo a la promoción y el apoyo de la lactancia materna. Sin embargo, algunas barreras subrayan la importancia del conocimiento previo para una comprensión completa del contenido.

## INTRODUCTION

Significant learning transforms knowledge, enabling students to grasp concepts and act autonomously throughout their professional journey^(1)^. This understanding implies the implementation of pedagogical practices that promote learning, especially when intending to use technological resources that contribute to the professional training of health students^(2)^. Research involving students from Portugal showed that the applied pedagogical model considered using technological resources, such as audiovisuals, due to their positive effects on the self-learning of postgraduate students^(3)^. This type of technology holds educational potential because of its visual and filmic language, aiding in both the acquisition of scientific knowledge and the autonomy of students in their cognitive constructs. Thus, audiovisual technologies can serve as instruments associated with pedagogical practices, mediating the teaching-learning process^(4, 5)^.

Among the scientific content to be mastered by health students is lactation physiology, which focuses on developing evidence-based breastfeeding (BF) management^(6)^, as well as counseling women to achieve their breastfeeding goals^(7)^ and meet sustainable development objectives^(8)^. However, lactation physiology includes a set of complex and abstract contents^(9)^, related to hormones and their role in producing breast milk^(10)^. Consequently, educational technologies can be effective mediating tools for teaching and learning, such as videos on BF content^(11)^, motivation^(12)^, and self-efficacy^(13)^ in breastfeeding. Investing in technologies addressing this topic highlights the importance of using technologies to mediate BF promotion content.

From this perspective, methodological studies have been undertaken to create and validate with experts a video clip^(14, 15)^ that promotes the learning of lactation physiology, grounded in the Knowledge Translation Model^(16)^. Therefore, considering the validated tool, the research problem lies in determining whether the educational technology is appropriately tailored for a specific target audience, such as undergraduate health students in the current study. This aligns with the contemporary challenge, identified in a scope review, of applying new knowledge to reduce the gap between evidence and clinical practice. This challenge involves engaging end-users and stakeholders, and considering the context-specificities where the tool will be used^(17)^.

The hypothesis is that the video clip is suitable for pedagogical use with undergraduate health students, and that it is essential to consider the barriers in the local context for its continued use. To support this hypothesis, evaluating the video clip is necessary, and there are various methods for evaluating educational technologies (ET) that can be developed across different knowledge areas^(18)^. These evaluations should consider the social dimension, requiring an assessment of the target audience’s understanding^(19)^, and should ideally be conducted in a manner that promotes audience engagement, enhancing the tool’s continued use^(20, 21)^.

## OBJECTIVES

To assess the suitability, facilitators, and barriers to using a video clip in teaching lactation physiology to undergraduate health students.

## METHODS

### Ethical Aspects

This study is part of the thesis entitled “Evaluation of a Video Clip for Learning Lactation Physiology by Undergraduate Health Students”^(22)^, approved by the Human Research Ethics Committee of the Federal University of Santa Maria, in accordance with Resolution No. 466/12 of the Ministry of Health^(23)^.

### Study Design, Location, and Period

A cross-sectional study was conducted with online data collection, using tools provided through the Student Portal, with assistance from the Data Processing Center (CPD) of the higher education institution where the study was developed, located in Southern Brazil. Data collection took place between May and September 2021.

### Sample, Inclusion, and Exclusion Criteria

There were 2,475 students enrolled in the eight undergraduate health courses at the institution: Nursing, Pharmacy, Physiotherapy, Speech Therapy, Medicine, Nutrition, Dentistry, and Occupational Therapy. It was determined that for the technology to be classified as adequate, 55% of the sample needed to rate it as good^(24, 25)^. Based on the mentioned population, a 10 percentage point margin of error, and a 95% confidence level, at least 92 participants were required in the sample. This calculation was performed using the WINPEPI 11.65 program^(26)^. Inclusion criteria considered were undergraduate health students from a public higher education institution. All health courses were included due to the multi-professional nature of breastfeeding support, aligning with global policies for the promotion, protection, and support of breastfeeding, which necessitate training on the topic. No criteria were established regarding academic aspects and performance. The final sample consisted of 88 students, without course stratification.

### Study Protocol

This study is part of a broader Knowledge Translation project, based on a model developed in Canada, known as Knowledge Translation^(16, 20, 21)^. This project type proposes the application of evidence in various care practice settings and comprises two cycles: a creation cycle, where evidence synthesis is developed and technologies can be created, and an application cycle, which encompasses six phases: adapting knowledge to the local context; evaluating barriers and facilitators for knowledge use; selecting, tailoring, and implementing interventions; monitoring knowledge use; evaluating the impact; and sustaining knowledge use^(16, 20, 21)^.

In the current study, where the video clip was evaluated by students, one phase of the application cycle of the model was addressed: identifying barriers and facilitators to knowledge use. Notably, the video clip was validated by experts in two preceding methodological studies in the creation cycle of the knowledge translation model: the first to create and validate musical content^(14)^ and the second to create and validate imagery content^(15)^. Its creation was a commitment to translating the complex and abstract knowledge of lactation physiology, aiming to introduce this content and mediate the target audience’s learning to complement the actions of promoting and supporting breastfeeding. The video clip has a duration of 2 minutes and 34 seconds.

As a product of the knowledge translation project, the video clip was developed under the researchers’ guidance by professionals from various fields working in the Educational Technology Coordination (CTE) and the Music Department, located at the institution affiliated with the researchers. The final product, titled “Lactashow: The Lactation Cycle,” was registered and is freely accessible at: https://www.youtube.com/watch?v=dhiUfNXu7AE.

To meet the study’s objective of assessing the video clip’s suitability for the target audience of university health students, the Assistive Technology Assessment Instrument (IATA)^(24, 25)^ was utilized, and open-ended questions were formulated to evaluate facilitators and barriers.

The instrument^(24, 25)^ used in this study was originally designed to evaluate educational technology (ET) for an audience with visual impairments. Given that the same instrument might not be reliable under different conditions, such as the population it is applied to^(27)^, the internal consistency of the instrument in the student sample was assessed. A Cronbach’s alpha coefficient of 0.93 indicated good reliability of the instrument’s items for this population.

Consequently, through meetings with the CPD team, a weekly schedule was organized to send electronic correspondence with invitations to all students enrolled during the data collection period. The Undergraduate Course Coordination of the institution also contributed by sending an invitation email to the classes. Moreover, the project team promoted the research on social networks.

Initially, students received the sociodemographic characterization instrument for the target population. After their first access to the video clip, the IATA^(24, 25)^ was made available, comprising fourteen questions distributed across four attributes: interactivity; objectives; relevance and effectiveness; and clarity.

Five open-ended questions were included to capture aspects that participants deemed positive or negative, suggestions for adapting the video clip to the local context, opinions on using audiovisual technologies in the learning process, and technical aspects of access across different devices. The instrument underwent a pilot test with students affiliated with the Research Group, leading to necessary adjustments in the open-ended questions.

### Analysis of Results and Statistics

The Assistive Technology Assessment Instrument (IATA) allows for the evaluation of each attribute, with scores ranging from 0 to 2, defined as follows: inadequate (when the technology does not meet the item’s definition), partially adequate (when the technology partially meets the item’s definition), and adequate (when the technology fully meets the item’s definition)^(24)^. An attribute was deemed inadequate if the average score was 0; partially adequate if the average score ranged from 0.1 to 1; and adequate if the average score varied from 1.1 to 2^(25)^. The average of these attributes led to the overall classification of the video clip’s adequacy. Cronbach’s Alpha was computed to verify the internal consistency of the scale items, considering a value above 0.7 as ideal^(26, 27)^. The Mann-Whitney test was employed to examine the association between binary variables (gender, age, undergraduate course, possession of a course covering lactation physiology content, completion of such a course, and self-assessed prior knowledge of the content). The level of significance was set at 5% (p < 0.05).

Responses to the open-ended questions underwent categorization^(28)^. To maintain the confidentiality of participant identities in the presentation of qualitative data, a coding system was utilized, denoting each entry with the letter “E” followed by a sequential number.

## RESULTS

Eighty-eight undergraduate health students participated in the evaluation of the video clip. The majority were female (71.6%, n=63), and the predominant age group was 18 to 29 years (85.2%, n=75). Regarding their enrolled courses, Nursing had the highest incidence (28.5%, n=25), followed by Pharmacy (18.3%, n=16), Speech Therapy (12.5%, n=11), Occupational Therapy (10.3%, n=9), Medicine (9%, n=8), Nutrition (9%, n=8), Physiotherapy (7.9%, n=7), and Dentistry (4.5%, n=4). Most participants did not report any disabilities (94.3%, n=83) and indicated a high use of digital devices and the internet (99.1%, n=87).

The video clip was evaluated by the target audience of university health students as adequate in all attributes (Table 1). This indicates that the video clip promotes engagement in the learning process autonomously, as it can be accessed at any time by the student, according to their needs in clinical practice and academic life. For science, the suitability of an educational technology for the student target audience represents an advancement in the frontier of knowledge on the topic of breastfeeding (AM), as it translates the knowledge of lactation physiology, a content considered complex and abstract, yet essential for understanding the breastfeeding process.

**Table 1:** Description of the results of the evaluation of the video clip attributes by undergraduate health students, Santa Maria, Rio Grande do Sul, Brazil, 2021 (N=88)

Attributes	Adequate	n (%) Partially Adequate	Inadequate
Interactivity			
1.The content of the information is suitable for your needs.	63 (71.6)	22 (25)	3 (3.4)
2. Offers interaction, active engagement in the educational process.	65 (73.9)	20 (22.7)	3 (3.4)
3. Allows easy access to the presented topics.	68 (77.3)	17 (19.3)	3 (3.4)
4. Provides user autonomy in terms of operation.	65 (73.9)	21 (23.9)	2 (2.3)
Objective			
5. Stimulates learning about the covered content.	75 (85.2)	9 (10.2)	4 (4.5)
6. Encourages the learning of new concepts.	73 (83)	13 (14.8)	2 (2.3)
7. Enables you to seek information effortlessly.	71 (80.7)	14 (15.9)	3 (3.4)
8. Has an attractive presentation strategy.	66 (75)	18 (20.5)	4 (4.5)
Relevance and Effectiveness			
9. Provides adequate and necessary resources for its use.	69 (78.4)	18 (20.5)	1 (1.1)
10. Sparks your interest in using it.	61 (69.3)	26 (29.5)	1 (1.1)
11. Encourages behavioral changes in you.	49 (55.7)	33 (37.5)	6 (6.8)
12. Reproduces the covered content in different contexts.	59 (67)	26 (29.5)	3 (3.4)
Clarity			
13. Presents information in a simple manner.	68 (77.3)	18 (20.5)	2 (2.3)
14. Allows you to reflect on the covered content.	73 (83)	12 (13.6)	3 (3.4)

In this study, the video clip was evaluated as adequate, as were all its other attributes (Table 2). In the attribute of interactivity, the item “allows easy access to the presented topics” scored the highest. Regarding the objective attribute, the video clip was most highly rated for “stimulating learning about the covered content.” In relevance and efficacy, the highest score was for the video clip “providing adequate and necessary resources for its use.” Lastly, in the attribute of clarity, the item “enables reflection on the covered content” received the highest score in the evaluation of the video clip.

**Table 2:** Evaluation of the video clip attributes by undergraduate health students, Santa Maria, Rio Grande do Sul, Brazil, 2021 (N = 88)

Attributes	Average (SD)	Median (IQR)
Interactivity	1.71 (0.43)	2 (1.50-2.00)
Objective	1.77 (0.42)	2 (1.75-2.00)
Relevance and Effectiveness	1.64 (0.38)	1.75 (1.50-200)
Clarity	1.77 (0.43)	2 (1.50-2.00)
Overall Adequacy Rating	1.72 (0.36)	1.87 (1.57-2.00)

*SD – Standard Deviation; IQR – Interquartile Range.*

There was no significant difference in the comparison of attributes between the independent sociodemographic variables and prior knowledge of lactation physiology (Table 3).

This indicates to educators the suitability of the technological product for their pedagogical practice, as they can use the video clip without it being dependent on academic profile or performance context for application in a learning environment. Considering that there are other supporting technologies for this process, using these in a complementary manner in training could mediate learning to achieve the global goal of exclusive breastfeeding rates, which are currently below expectations.

**Table 3:** Independent variables associated with the categorical outcome of the evaluation of the video clip attributes by undergraduate health students, Santa Maria, Rio Grande do Sul, Brazil, 2021 (N = 88)

Median (IQR)	Interactivity	Attributes		Clarity	Overall Adequacy Rating
		Objective	Relevance and Effectiveness		
Sociodemographic Variables					
Gender	p=0.165	p=0.558	p=0.706	p=0.943	p=0.448
Male	1.75 (1.37-2.00)	2 (1.50-2.00)	1.75 (1.50-2.00)	2 (1.50-2.00)	1.87 (1.44-1.97)
Female	2 (1.50-2.00)	2 (1.75-2.00)	1.75 (1.50-2.00)	2 (1.50-2.00)	1.87 (1.62-2.00)
Age (in years)	p = 0.406	p = 0.884	p = 0.854	p = 0.663	p=0.582
>18-29	2 (1.50-2.00)	2 (1.75-2.00)	1.75 (1.50-2.00)	2 (1.50-2)	1.87 (1.56-2.00)
>30	2 (1.62-2.00)	2 (1.62-2.00)	1.75 (1.50-2.00)	2 (1.25-2)	1.87 (1.56-2.00)
Prior Knowledge of Lactation Physiology Content					
Course includes the content	p=0.946	p=0.320	p=0.657	p=0.414	p=0.982
Yes	2 (1.62-2.00)	2 (1.50-2.00)	1.50 (1.50-2.00)	2.00 (2.00-2.00)	1.87 (1.56-2.00)
No	2 (1.51-2.00)	2 (2.00-2.00)	1.75 (1.50-2.00)	2 (1.50-2.00)	1.87 (1.58-2.00)
Completed this course	p=0.504	p=0.517	p=0.519	p=0.827	p=0.896
Yes	2 (1.75-2.00)	2 (1.62-2.00)	1.50 (1.50-2.00)	2 (1.75-2.00)	1.87 (1.59-2.00)
No	2 (1.50-2.00)	2 (1.81-2.00)	1.75 (1.50-2.00)	2 (1.50-2.00)	1.87 (1.56-2.00)
Has knowledge of the content	p=0.946	p=0.593	p=0.688	p=0.415	p=0.594
Yes	2 (1.37-2.00)	2 (1.75-2.00)	1.75 (1.25-2.00)	2 (1.50-2.00)	1.94 (1.53-2.00)
No	2 (1.50-2.00)	2 (1.50-2.00)	2 (1.50-2.00)	1.75 (1.50-2.00)	1.87 (1.69-2.00)

*IQR – interquartile range.*

**Figure 1:**
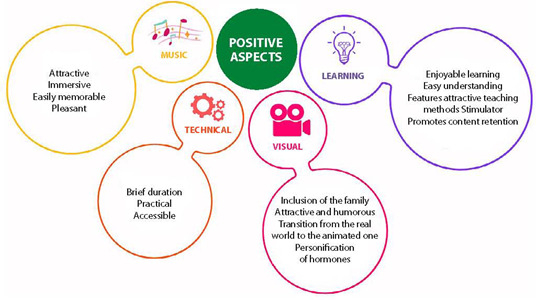
Facilitators for the Use of the Video Clip by Undergraduate Health Students, Santa Maria, Rio Grande do Sul, Brazil, 2021

In the qualitative assessment, 31 participants indicated facilitators for use, such as the attractiveness and retention promoted by the musical and animation features, technical aspects of ease of access, learning aspects, among others (Figure 1).

Students noted aspects they considered positive about the music:

*The music is an excellent tool for attraction.* (E5)

*It has the capacity to engage.* (E10)

*It is easily memorable.* (E10, E12)

*It remains memorable even after just one listen.* (E25)

*The music has a pleasant quality.* (E25)

In terms of the positive aspects of the visual content, students highlighted:

*The inclusion of the family in the breastfeeding process depicted at the start of the video.* (E7)

*The attractive and humorous imagery.* (E14, E15, E24, E31)

*The transition from the real world to the animated one.* (E25)

*The personification of hormones as entities that contribute to the functioning of lactation physiology.* (E26)

Regarding the use of audiovisual technologies in the learning process, students observed that the video clip:

*Facilitates enjoyable learning.* (E1)

*Is easy to understand.* (E1, E9, E11, E13, E20)

*Features attractive teaching methods.* (E7, E13, E22)

*Stimulates learning in various ways.* (E1, E11, E25)

*Effectively combines audio and visual elements to enhance content retention.* (E9, E10, E11, E14, E17)

*Aligns with the new teaching paradigm, utilized in a hybrid approach.* (E8, E22, E24, E29, E31)

As for the technical aspects, students identified the following positives:

*The brief duration of the video.* (E9)

*Accessibility to a diverse audience* (E12, E15), *including those with hearing impairments due to the inclusion of subtitles.* (E10)

*Ease of access* (E25), *including availability on social media platforms.* (E15)

*The video does not require a highspeed internet connection for viewing.* (E20)

It was evident that the musical and imagery content, which comprise the animation of the video clip, was considered attractive by the students, indicating the potential for revisiting the content. The students believe that the characters in the video clip, representing the hormones involved in lactation physiology, concretize the content and facilitate learning, and that the musicality aids in the retention of knowledge. Regarding technical aspects, the availability of open access and the short duration facilitate its use in teaching practices.

**Figure 2:**
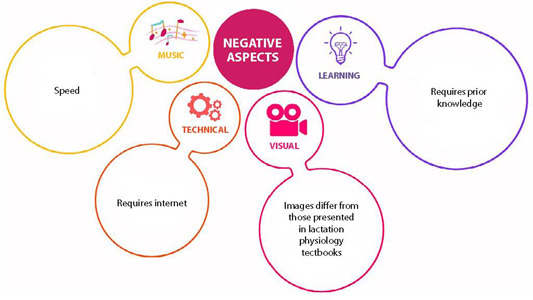
Barriers to the Use of the Video Clip Reported by Undergraduate Health Students, Santa Maria, Rio Grande do Sul, Brazil, 2021.

Additionally, participants reported barriers to use, such as the speed of the music and the need for prior knowledge (Figure 2).

Students identified technical issues such as access difficulties for those without internet (E29) and considered certain aspects of the music and visual content as negative:

The music is too fast considering the amount of information in the lyrics. (E6, E24)

The speed at which the images change [...] is a factor that makes understanding difficult. (E6, E26)

Difficulty in paying attention to both audio and visual elements at the same time. (E6)

Images differ from those presented in lactation physiology textbooks. (E15)

Regarding the learning process, students noted that:

Prior knowledge is necessary. (E3)

Just watching the video makes it confusing to understand the action of each hormone. (E26)

The issues related to barriers allow us to indicate the music video as a mediator of learning that requires prior knowledge and more than one approach to the object of study. There was no indication from the students to adapt the music video to the local context.

## DISCUSSION

The video clip was deemed appropriate by the participants, achieving an overall average of 1.72 across four attributes: interactivity, objective, clarity, and relevance. These positive outcomes indicate acceptance of the video clip^(29)^ and demonstrate the feasibility of using this technology in teaching, thereby promoting student autonomy, as the video clip can be accessed freely.

In terms of the interactivity attribute, the video clip, considering both its audio and animation, meets the students’ needs for engagement in the educational process, facilitating easy access to topics in lactation physiology. A similar study^(24)^ evaluated an app for teaching and learning the Portuguese language for the hearing impaired^(30)^, also using the same evaluation instrument, and deemed it appropriate in the interactivity attribute^(25)^. This outcome suggests that the app provides users with autonomy in learning a second language^(30)^. Developing and incorporating interactivity in any technology acknowledges its potential to foster active learning.

The appropriateness in the objective attribute suggests that the video clip effectively supports the learning of the physiological process of human lactation. Another study, evaluating technology in two countries, also observed positive results in this attribute among Brazilian and Portuguese participants^(25)^. The study involving the hearing impaired also found the technology appropriate in the “objective” attribute^(30)^. For technological learning to be impactful, it should integrate seamlessly into the individual’s cognitive structure in a non-arbitrary way^(1)^. Understanding physiology is vital to comprehend breastfeeding management, as hormone interactions positively influence milk maintenance and production. Thus, this learning is crucial in offering support aligned with theoretical content on milk production.

Moreover, the video clip’s engaging strategy, which includes the use of music and animation to depict hormones involved in lactation physiology, was rated as suitable. Facilitators for using the video clip, as indicated by the participants, included its pleasant musicality, audiovisual aspect, appeal, and ease of understanding, showcasing its potential for usability. The video clip’s capacity to engage students in the learning process through its musical elements aligns with a study where researchers noted that music makes content more relatable, engaging students and encouraging them to develop critical and reflective thinking^(31)^. Videos and music are regarded as motivational tools in the teaching-learning process, particularly when used in conjunction^(32)^.

The clarity attribute pertains to how well the video clip’s content meets students’ needs in comprehending a complex and abstract subject, signifying that this educational technology facilitates understanding of the physiological process of human lactation. A parallel study that applied the same evaluation method for assistive technology also reported high scores in the clarity attribute^(25)^. When material is easily comprehensible, it possesses substantial potential. For learning to be significant, learners need to meaningfully connect with the material, logically integrating it into their cognitive framework^(1)^. The perceived clarity of the video clip is remarkable, especially given the complexity of lactation physiology, which involves hormone interactions, mammary tissue development, and breast milk production, and is an abstract concept that is challenging to grasp due to its intangible nature.

The video clip was considered appropriate by participants, achieving a global average of 1.72 across four attributes: interactivity, objective, clarity, and relevance. These positive results indicate acceptance of the video clip^(29)^ and the feasibility of using this technology in teaching, as it promotes student autonomy due to the free accessibility of the video clip.

The assessment of relevance showed that the resources of the video clip are sufficient to spark students’ interest in its use for learning lactation physiology. This is consistent with an assessment conducted by Brazilian and Portuguese participants, where both groups achieved an average of 1.65 in the same attribute^(25)^. However, the results suggest that while the video clip is adequate, it may not fully stimulate behavioral changes in research participants, as only 55.7% rated this attribute as adequate. This implies that while the video clip can be utilized, it is advisable to combine it with innovative pedagogical practices and the educator’s experience in an active learning process that can influence discussions of real professional practice situations where this knowledge will be applied.

Health students must comprehend the physiological processes involved in lactation to effectively support breastfeeding (AM) in professional practice. Yet, some health education programs do not include AM topics in their curricula. A study in the United States indicated that 71% of pediatric and obstetric medical professionals feel insecure about advising on AM, and many still recommend weaning in unnecessary situations. Moreover, these professionals are often unaware of the physiological processes that occur from gestation to milk “let-down”^(33)^. It is crucial to note that AM guidance should start in prenatal care and be reinforced during the childbirth process. Inadequate or incomplete guidance during prenatal care, coupled with a lack of support during labor and delivery, increases the likelihood of early weaning^(34)^. Thus, it is essential for health students to understand the physiological processes of lactation to effectively participate in multidisciplinary teams in various clinical settings.

Participants identified as a facilitator the potential of the video clip to adapt to a hybrid teaching model. This is particularly relevant in the context of the Covid-19 pandemic experienced by students during the data collection period of this study. With social distancing recommendations, the academic community had to transition from entirely in-person to remote classes^(35, 36)^. When organized and executed properly, hybrid teaching can lead to meaningful learning.

Regarding the barriers to using the evaluated technology, the speed of the music in relation to the large amount of information presented was seen as a potential limitation in the learning process. In addition, participants indicated the necessity of prior knowledge^(37)^ to understand the content, suggesting that the video clip might act as a subsumer, in line with Ausubel’s learning theory^(1)^.

Evaluating the barriers was essential to ensure the effective use of this technology in its intended context. The analysis of the two barriers reported by participants is consistent with the theory of meaningful learning: for learning to be effective, it is vital to understand what the learner already knows so that the new content has logical significance^(1)^ This underscores the notion that the use of technologies by educators enhances students’ comprehension of content, a positive finding also demonstrated in other studies^(38, 39, 40)^. It is critical to consider the identified barriers in different local contexts to ensure the continued use of the technology.

Given the evaluation of the need to incorporate other audiovisual technologies into the learning process, we emphasize the importance of continuous monitoring of the use of the technology in this study, to understand its impact in the context where it is implemented. This approach is in line with the Knowledge Translation Model, highlighting the researcher’s role in navigating the creation-action cycle phases, aiming to sustain the tool’s use in the intended context.

### Study limitations

Firstly, the low participation rate among students, attributed to the Covid-19 pandemic context and the subsequent transition to remote classes, impacted the study’s adherence. Despite using online questionnaires to facilitate remote access, student responses fell below expectations. Additionally, a critical limitation was the exclusion of students with visual or auditory disabilities from data collection, since the video clip was not developed with accessibility features for these groups. As a result, the findings regarding the video clip’s suitability for undergraduate health students are not generalizable to these specific populations. Therefore, while the study’s findings are relevant, they should be interpreted with caution due to these significant constraints.

### Contributions to the Fields of Nursing and Education

The study’s findings can contribute to the recognition of systematic technology evaluation by end-users, pointing to possibilities for local context adaptations and usability enhancements. The study also aids in guiding the adoption of hybrid teaching strategies in health education, particularly in breastfeeding education.

Notably, even though technological prospecting is a long-term endeavor, the research group sent a communication with the access link to this product to the Brazilian Nursing Association (ABEN Nacional), requesting its broad dissemination among Brazilian universities to support evidence-based and hybrid teaching. Furthermore, a promotional poster with the link and QR Code for accessing the video clip was sent to services connected to the Regional Health Coordination. This initiative to promote breastfeeding also aimed to support evidence-based clinical practice with technology and innovation. In the broader knowledge translation project, maintaining the use of this knowledge product is a key strategy.

## CONCLUSIONS

The video clip is an interactive, objective, clear, and relevant tool, suitable for use in pedagogical practice with undergraduate health students. This educational technology, which translated complex and abstract knowledge of lactation physiology, can serve as a learning strategy that enhances hybrid teaching in training.

The positive assessment of its suitability and facilitators, such as attractiveness, memorability promoted by the video clip, and ease of access, highlight the tool’s potential to introduce lactation physiology content and facilitate learning. This complements the actions of promoting and supporting breastfeeding, enabling autonomous professional practice.

The target audience’s perception that there is no need to adapt the video clip to the local context suggests the potential for applying this educational technology in undergraduate health courses at Public Higher Education Institutions.
